# Sociodemographic and morbidity characteristics of people on long-term sick leave

**DOI:** 10.1186/s12889-022-14665-w

**Published:** 2022-12-02

**Authors:** Corina Oancea, Rodica Simona Capraru, Ana Maria Alexandra Stanescu, Despina Mihaela Gherman

**Affiliations:** 1 University of Medicine and Pharmacy “Carol Davila”, Bucharest, Romania; 2 The National Institute for Medical Assessment and Work Capacity Rehabilitation, Bucharest, Romania

**Keywords:** Sick leave, Long-term care, Risk factors, Sociodemographic factors, Morbidity

## Abstract

**Background:**

Certifying long-term sick leave and coordinating complex rehabilitation programs are essential activities of social insurance doctors. These doctors have a role in preventing the decreased work capacity of employees that may lead to leaving the labour market and the transition of these employees to other social insurance benefits, such as a work disability pension.

**Objectives:**

Analysis of long-term sick leaves (over 183 days) to identify risk factors and population groups with low potential for work capacity rehabilitation.

**Method:**

We conducted a cross-sectional study between September 2019 and September 2020. The information was collected from the National Institute of Medical Assessment and Work Capacity Rehabilitation Bucharest registers and the EXPMED application. The data were statistically analysed using PSPP software.

**Results:**

The highest rehabilitation percentage was achieved in cases of traumatic injuries (73.17%), followed by musculoskeletal diseases (70.06%). We noticed lower recovery in cases of nervous system diseases (50.56%) and cardiovascular diseases (44.23%). In the group that summed up the other pathologies, the recovery percentage was 58.37%. People who regained their work capacity were significantly younger (mean age 47.87 y ± 8.93) than those who turned to other forms of social benefits, such as a disability pension or an old-age pension (mean age 53.16 y ± 8.43).

**Conclusion:**

Most of the subjects (72%) regained their work capacity and did not need a disability pension. We identified the sociodemographic and morbidity characteristics of people on long-term sick leave along with target groups requiring intensive intervention measures.

**Supplementary Information:**

The online version contains supplementary material available at 10.1186/s12889-022-14665-w.

## Background

Absenteeism from work produces measurable effects on labour productivity and generates additional costs for social security systems. Sick leave is a benefit granted by law to employees who are physically or mentally unable to carry out their professional activity for variable periods depending on the nature and severity of their health condition [1, 2].

In almost all European Union countries, employer first grants sick leave benefits for a variable period (depending on the country) and later by a social security system. Member States can be divided into two groups concerning the duration of sick pay. In the first group, the benefit for a short period of sick leave time is paid by the employer: from two paid days in Lithuania and three in Bulgaria to a maximum of 2 weeks in the Czech Republic, Estonia, Hungary, Finland, Latvia, Slovakia, Spain and Sweden [3, 4]. In the second group of countries, sick pay by the employer is much longer, ranging from more than a month in Austria (6 to 12 weeks) to a 104-day maximum in the Netherlands and 180 days in Italy and Croatia (42 weeks). The current legislation in Romania stipulates that the amount corresponding to the first five working days of sick leave be paid by the employer and for the following days to be paid from the Unique National Health Insurance Fund (FNUASS-Romanian abbreviation) [5].

At the end of the period that the employer pays the sick benefit, allowances are provided by the social security systems in all Member States. The maximum length of paid sickness benefits varies widely between countries, ranging from 22 weeks out of 9 months in Denmark to 3 years in Portugal. Slovenia and Bulgaria are the only countries where sickness benefits are granted for an unlimited period. Social insurance physicians may consider ending sick leave or transitioning to another social security benefit (e.g., work disability pension).

In Romania, the maximum duration of sick leave is up to 183 days, with the possibility of extension up to 273 days in certain situations, both for common diseases or injuries outside work, as well as for work accidents and occupational diseases. The temporary incapacity for work is accepted for a longer period, between 1 year and a year and a half, in special cases (such as cancer, AIDS, tuberculosis, and some cardiovascular diseases) [5].

Social security legislation for temporary work incapacity provides cash benefits to compensate for the loss of income due to illness. This financial support during periods of temporary incapacity for work is assigned for medical treatment and work capacity rehabilitation. Long-term sick leave is a major concern for all health insurance systems. Returning to work after temporary work incapacity must follow all the necessary steps for the safe reintegration of the worker while paying attention to the proper management of the period of temporary work incapacity [6]. Access to appropriate medical treatment as soon as possible and suitably modified work for a position that maximizes skills and experience are important parts of prevention in health care [7].

The activity of social insurance physicians regarding the evaluation of the temporary incapacity for work involves a comprehensive approach focused on assessing the remaining abilities and prognosis of work capacity rehabilitation in cases of long-term incapacity for work (91–273 days of sick leave) for all medical conditions [5, 8]. The main objective is to avoid work disability by early identification of the optimal time range in which a maximum medical improvement could be obtained, keeping the patient’s ability to successfully return to work in a safe, productive and competitive way.

We assume that a significant step in correctly managing of these cases would be for social security physicians to more responsibly assume their leadership role in supervising long periods of temporary work incapacity, together with other health care providers, such as attending physicians or general practitioners. The recommendations for establishing the optimal period of temporary incapacity for work include successive evaluations based on the systematic functional evaluation for the diagnosis every 30 days while staying within the maximum period provided by law.

The prognosis of work capacity rehabilitation is estimated according to the classical clinical and functional parameters while considering contextual factors.

The following strategy is in use:

1. The prognosis of work capacity rehabilitation is considered favourable if medical rehabilitation is obtained within the first stage of temporary work incapacity (first 90 sick days). This is the case for mild functional impairment, defined by improving specific functional parameters. Return to work is determined if the person performs light/sedentary work that is carried out under adequate working conditions. Another 30 days of sick leave will be approved until normal functioning is obtained in case of more demanding professional activities or comorbidities that may increase the risk of more severe forms of the disease with poorer functional status.
2. If the rehabilitation program from the first stage of temporary work incapacity (first 90 sick days) does not bring enough improvement (suggested by the persistence of abnormal functional parameters), the social insurance physician may request a new medical evaluation. Depending on the patient’s condition, an additional period of temporary work incapacity will be approved, or an assessment of the remaining work capacity will be recommended.
3. If optimal functioning cannot be achieved during the maximum period of temporary work incapacity provided by law, work capacity assessment will be indicated, according to current procedures. This situation is characteristic of disabling functional impairments when, despite active monitoring, the course of the disease is slowly unfavourable, the patient remains symptomatic, and the functional parameters do not significantly improve.
4. For severe medical conditions with poor prognoses during the prolonged period of sick leave, the minimum time necessary to complete the procedure for granting a degree of work disability will be approved.

Several determinant factors for work capacity rehabilitation have been described in the literature: sociodemographic, occupational or factors related to social security systems and policies [9–15]. Many extensive studies come mainly from Nordic countries, where a coordinated system of good practices works on the monitoring of absenteeism due to medical causes (temporary incapacity for work), prevention and the implementation of measures to promote early return to work and job retention [16–18].

The duration of sick leave is often viewed to reflect the severity of the condition. “Severity rate” is a term representing the mean number of sick-leave days/person and is one of the most common ways of measures used in sickness absence research [19]. The majority of sickness absence is generally attributed to sickness or incapacity, but other reasons were listed: macro level factors (e.g. sickness certification practices, taxation, pensionable age, social attitudes, unemployment), organisational level factors (e.g. working conditions, job demands, workforce availability, personnel policies) and individual level factors (e.g. age, sex, occupational status, length of service, job satisfaction, personality, family, responsibilities, social support) [18, 20].

Having a chronic disease affects work participation negatively; people with a chronic disease are less often employed [21]. Work participation is influenced by personal factors (e.g., age, education, gender) and work-related factors (e.g., heavy manual work or work environment) [22]. Encouragement and early intervention in targeted subgroups of workers are important factors, since the longer the sickness absence lasts, the less likely people are to return to work [21].

Previous research has shown that other factors (disease-generic factors) could influence the work participation of patients with various diagnoses besides disease-related factors. Personal and environmental factors, such as functional impairment, heavy manual work and female gender, were associated with work disability [23].

A systematic review found age and gender among work participation’s most commonly reported factors. Older age, lower educational levels or race were negatively associated with work participation. On the contrary, higher socioeconomic status was positively associated with work participation. Other factors were reported to be associated with work rehabilitation: comorbidity, living in an urban area, workplace environment and financial considerations [21].

One important mission of social insurance physicians is to contribute to better management of temporary work incapacity for different pathologies, from 91 to 273 days of sick leave. Specific legislation must be applied, however, knowledge about predictive factors for long-term sickness absence and work disability pension is limited.

## Aim

This study aimed to analyse long-term sick leave (over 183 days) to identify the risk factors and population groups with low potential for work capacity rehabilitation characteristics in Romania. To our knowledge, no such study has been performed until now.

The following research questions will be addressed: a) What are the pathologies in which there are the most numerous requests for long term sick leave? b) What are the pathologies in which the largest number of days of sick leave is requested? c) What are the determining factors for a longer sick leave? d) What are the pathologies in which rehabilitation is most often achieved? e) What are the determining factors for rehabilitation?

## Methods

### Data

We analysed the long-term sick leave (over 183 days) certified by The National Institute of Medical Assessment and Work Capacity Rehabilitation Bucharest (INEMRCM-the Romanian abbreviation). The procedures are established by the National House of Public Pensions (CNPP-the Romanian abbreviation) in relation to the disease progression and the results of the individualised rehabilitation program. For this purpose, we conducted a cross-sectional study between September 2019 and September 2020. A total of 3889 sick leaves were studied, 1306 in 2019 and 2583 in 2020, with an average of 299 sick leaves/month.

### Variables

Few sociodemographic factors were available: age, gender and county of residence. The counties were grouped into eight major regional divisions, nonadministrative units with a role in collecting regional statistics. Their geographical position in the country names them: (1) Northeast, (2) Southeast, (3) South, (4) Southwest, (5) West, (6) Northwest, (7) Centre, and (8) Bucharest-Ilfov. The analysis of the degree of GDP in each developing region in Romania from 1995 to 2010 highlighted that the South and North regions are two regions with a low level of economic development; in contrast, the Bucharest-Ilfov and Western and Northwest regions have a higher level of economic and social development than the other regions (Table 1) [24–26].

**Table 1:** Number of requests depending on the development regions of Romania

Regions	Name	Number of requests (%)	GDP (% from EU average)
1	Northeast	426 (3%)	36
2	Southeast	303 (6%)	52
3	South-Muntenia	729 (8%)	46
4	Southwest-Oltenia	417 (11%)	42
5	West	283 (14%)	60
6	Northwest	198 (17%)	51
7	Centre	481 (19%)	54
8	Bucharest-Ilfov	1052 (22%)	139
Total	Romania	3889 (100%)	58

Medical cause for absence due to sickness (illness or injury) was available in 58% of cases, and the number of days granted in each case was specified (number of absence days/person). To analyse the medical cause, clinical diagnosis was coded according to ICD-10, the 10th revision of the International Statistical Classification of Diseases and Related Health Problems.

In 87% of cases, we also had information about the client’s condition at the end of the sickness period. This outcome was dichotomised as either returning to work or not Three categories were established for work capacity: fully recovered and resumed professional activity (preserved work capacity), eligible to receive a work disability pension (permanent incapacity for work) or old-age pensioners.

The number of days of sick leave or the impairment of work capacity decided in each case was carried out in accordance with current guidelines and regulations [5, 8, 27–29].

Depending on the number of requested days off from work due to illness, three categories of time off paid were analysed: less than 30 days (most for 30 days), between 31 and 60 days (most for 60 days) and over 61 days (most for 90 days).

Work disability was classified in relation to the degree of loss of work capacity according to law no. 263/2010 on the unitary system of public pensions [27]:

- 1st degree, characterized by total loss of work capacity and self-care, persons need daily assistance for basic activities;
- 2nd degree, characterized by the total loss of work capacity, while maintaining self-care ability (full work disability pension) and
- 3rd degree, characterized by the loss of at least half of the work capacity, the person being able to perform a professional activity part-time (partial work disability pension).

### Statistical analysis

Using descriptive statistics, data were presented as the number, percentage (%), mean, and standard deviation (SD). All sociodemographic and medical parameters were compared between groups. We used the chi-square test for nominal and categorical variables (e.g., gender, county of residence, diagnosis, and condition at the end of sickness absence) and an independent-samples t-test for numeric variables (e.g., age and number of days of sick leave).

The strength and the direction of the relationship between different variables was studied by correlation analysis. Multiple linear regression was used for the continuous dependent variables (e.g. length of sick leave) and multiple logistic regression for dichotomous dependent variables (e.g. return to work or not).

The statistical significance (*p*-value) was established at 0.05, as is the convention, and parameters were estimated for a 95% confidence interval. The data were processed with PSPP software.

## Results

### Sociodemographic situation

The subjects mainly were men: 2140 (55%) men vs 1749 (45%) women. The mean age of the group was 49.45 ± 9.15 years, with generally lower ages among women: the average age of women/men: was 48.35 ± 8.48 vs 50.36 ± 9.57; *p* < 0.001.

The group included all people who requested more than 183 days of sick leave in the specified period. The applications came from all counties in Romania, and they were grouped according to their region (Table 1).

### Number of requests and days needed for recovery

The average number of sick days over 183 days for various pathologies was 64.81 ± 27.65. There were also calculated the means for each category of time off paid as follows: 24.40 ± 8.91 days for less than 30 days (886 requests); 54.25 ± 9.68 days between 31 and 60 days (950 requests) and 88.79 ± 4.89 days over 61 days (2053 requests). Most requests for absence due to sickness were determined by musculoskeletal disorders (ICD-10 code M00-M99–46.28%), followed by traumatic injuries (ICD-10 code S00-T98–28.64%). In third place were the diseases of the circulatory system (ICD-10 code I00-I69–7.83%), and diseases of the nervous system (ICD-10 code G00-G69) occupied fourth place (4.62%). Other pathologies accounted for 12.63%, *p* < 0.001 (Table 2). In this last category, we grouped all other pathologies for which fewer than 100 applications were registered during the study, ranging from 1 application (0.04%) for external causes of morbidity (ICD-10 code V01-Y98) to 73 applications (3.21%) for diseases of the digestive system (ICD-10 code K00-K95); 53 applications (2.33%) were registered for mental, behavioural and neurodevelopmental disorders (ICD-10 code F00-F99).

**Table 2:** Distribution of requests and rehabilitation according to ICD-10

ICD-10	Nervous system (G00-G99)	Circulatory system (I00-I99)	Musculoskeletal system (M00-M99)	Injury (S00-T88)	Other pathologies
% of requests	4.62%	7.83%	46.28%	28.64%	12.63%
No. of days	68.19 ± 26.68	61.80 ± 28.75	69.54 ± 26.03	68.80 ± 25.07	52.08 ± 29.57
Rehabilitation (%)	50.56%	44.23%	70.06%	73.17%	58.37%

The highest average number of days was due to musculoskeletal pathology, 69.54 days for one case, followed by traumatic pathology, with an average of 68.80 days for one case; *p* < 0.001. The average number of approved days for the pathology of the nervous system was 68.19 and occupied third place. Diseases of the circulatory system were in fourth place, with an average number of 61.80 days/case (Table 2). In the case of common mental diseases, a low number of sick days was approved (34.91 ± 23.14 days).

Men required more days to recover than women (66.03 ± 26.92 vs 63.29 ± 28.46; *p* = 0.003). Additionally, a higher number of days of sick leave in the last year was used by those who retired (71.94 ± 25.04 vs 65.41 ± 27.31 days; *p* = 0.012).

### Social and economic impact of long-term sick leave

**Fig. 1:**
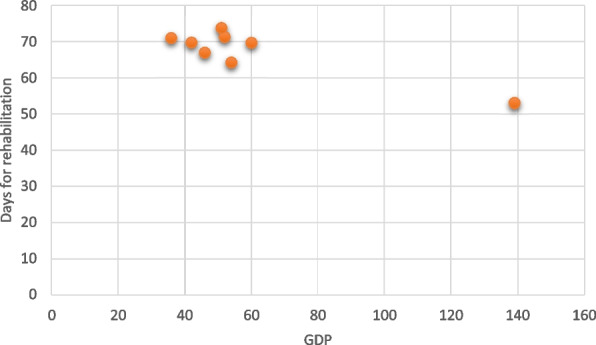
Correlation between the number of sick days needed for rehabilitation and the social and economic regional development level

We investigated the relationship between the number of days needed for rehabilitation and social and economic factors known for their impact on health and access to medical care. The number of days needed for rehabilitation was strongly correlated with the wealth and opportunities of the region of origin. Correlation analysis showed a negative correlation between the number of days needed for rehabilitation and the regional position in the economic ranking, in the sense that the number of days requested decreases with the increase in the level of social and economic regional development (*r* = − 0.89, *p* = 0.003); thus, persons from wealthier regions took significantly fewer days for rehabilitation (Fig. 1).

Statistical significance for the variables associated with the length of sick leave (regional development, clinical diagnosis, and retirement status) was tested using multiple linear regression. The best coefficients were obtained from these parameters for regional community and clinical diagnosis. In conclusion, the people predisposed to using a longer period of sick leave are those with traumatic or musculoskeletal pathology and who come from poor socioeconomic regions (Table 3).

**Table 3:** Odds ratio (COR) and 95% confidence intervals (CI) for factors associated with longer sick leave

Variable	Significance (***p***)	COR (95% CI)
Regional development	< 0.001	2.48 (2.05–2.90)
Clinical diagnosis	< 0.001	10.29 (7.78–12.80)

### Data regarding rehabilitation

The highest percentage of rehabilitation was achieved in the case of traumatic injuries (73.17%), followed by musculoskeletal diseases (70.06%). We noticed lower recovery for nervous system diseases (50.56%) and cardiovascular diseases (44.23%). In other pathologies, the recovery percentage was 58.37%, *p* < 0.001.

People who regained their work capacity were significantly younger than those who turned to other forms of social benefits, such as a disability pension or an old-age pension (47.87 ± 8.93 vs 53.16 ± 8.43); *p* < 0.001. In contrast, the recovery rate was not significantly different between the two sexes (70.71% vs 72.70%).

At the end of the period of temporary incapacity for work, 72.43% recovered and resumed their professional activity, 21.35% benefitted from a work disability pension (permanent incapacity for work), and 6.22% received early or full retirement benefits.

According to the degree of work disability, 1.35% benefited from the 1st degree, 7.20% from the 2nd degree and 12.80% from the 3rd degree.

Analysis of the condition at the end of the period of temporary work disability (depending on the medical cause, i.e., according to ICD-10 codes) showed that the highest percentage of recovery was obtained in cases of traumatic injuries (73.17%), followed by musculoskeletal diseases (70.06%). A lower recovery was observed in diseases of the nervous system (50.56%) and circulatory system (44.23%). Other pathologies recovered in 58.37% of cases (*p* < 0.001).

We performed a binary logistic regression analysis to certify the independent contribution of each variable (age and type of disease) to predict a return to work (nominal dichotomous variable – return to work: yes/no), The data suggest that age and clinical diagnosis are the best indicators for resuming professional activity at the end of the sick leave period; in this case, persons were not classified as work-disabled or retired (Table 4).

**Table 4:** Odds ratio (COR) and 95% confidence intervals (CI) for factors associated with return to work

Variable	Significance (***p***)	COR (95% CI)
Age	< 0.001	1.07 (1.06–1.09)
Clinical diagnosis	< 0.001	2.30 (1.85–2.86)

The mortality rate was low (0.2%). In particular, eight patients died within the study period: two from circulatory diseases (strokes), two from severe musculoskeletal diseases and four from traumatic injuries (complex polytrauma).

## Discussion

Our study showed that older people did not frequently resume their professional activity and applied for other forms of social benefits. Age has been identified in many studies as a strong predictor for employee absenteeism [17, 30–32]. However, the relationship is not analysed in detail. It is unclear whether the higher prevalence of absenteeism among older employees is due to physiological functional decline or to the fact that young employees are preferred in the labour market. The influence of age is also estimated in terms of sick leave duration. Thus, older employees may have longer sick leaves than younger employees. We did not find any significant difference in this regard. Workplace demands, cultural factors, and ethical and moral standards were also mentioned as potential factors that may influence absenteeism at the workplace, differentiated by age groups and regardless of clinical diagnosis [17, 30].

Higher rehabilitation was found in traumatic injuries and musculoskeletal diseases. Other studies indicated a low risk for disability pension in injuries [33].

Longer sick leave absences were reported for musculoskeletal diseases and traumatic injuries. Other authors have found that most days of sick leave were taken in the musculoskeletal disease or external cause categories [34]. A low number of sick days was approved in cases of common mental disorders. They currently represent a major societal challenge in many European countries and are 1st regarding work disability in Romania according to the 2018 statistical data of INEMRCM; cardiovascular diseases follow them. It is estimated that rehabilitation is longer in this group of diseases. Thus, many of them are classified early as a degree of work disability after a short sick leave (less than 183 days). However, some patients will recover later after a variable period with a work disability pension.

Our analysis revealed that more than 95.94% of the requested sick leave days were confirmed. However, the practice has shown that this percentage also includes the situations in which it was not possible to meet the timing of current procedures, thereby requiring approval to cover periods justified more by bureaucracy and less by poor health.

Marmot M et al. also discussed the possibility that absence from work to be an indicator of some lack of functioning with potentially psychological, social or physical causes. They underlined that sickness absence rates cannot be used uncritically as health measures, giving the example of countries similar in health and socioeconomic level (e.g. Belgium and The Netherlands) but with different sickness absence rates that are likely to be due to differences in social security and other policies [35]. Longer sick leave durations in those coming from less developed regions was found. Other studies have also reported a strong correlation between patterns of mortality and disability and geographical areas of socioeconomic deprivation [36, 37]. Several European studies have reported social and economic status as significant factors influencing temporary incapacity for work. The causes seem to be related to living conditions, behavioural factors and lifestyle with a direct impact on health or unfavourable working conditions [36, 38]. In our case, this might have several explanations: people in poorer regions may have had more severe forms of illness, and sick leave days have been used not only for recovery but also as a refuge from dissatisfaction at work (in terms of salary, work schedule or duties) or to compensate for the cumbersome administrative process.

Finally, the costs of longer sick leave durations are not to be neglected. The gross daily amount of the allowance for temporary incapacity for work due to a common illness represents 75% of the average daily income. Considering that in Romania in 2021, the average gross salary was approximately 1166 EUR, the annual financial impact on the budget for only compensating long-term sick leaves was estimated at more than 40 million EUR/year. Many authors have shown that other costs should be added to this amount, such as health care expenditures, impacts on workers and their families or losses in productivity, which are difficult to estimate [39]. Other papers estimated that the indirect costs of work accidents and occupational diseases could be four to ten times greater than the direct costs. The ILO estimates that lost working time, workers’ compensation, interruption of production, and medical expenses could cost up to 4 % of the global GDP [40].

Several limits of the study should be considered. Due to an incomplete information system, limited data were reported. Significant social and professional information was lacking about marital status, children, level of education, profession, length of service, and the field of activity or income. Indirect and rough information regarding economic status was made from the perspective of subjects residing in the developing regions of Romania.

In some cases, diagnosis or the condition at the end of the sickness period was missing. We processed only the present data, such that the examined groups were smaller and the study’s accuracy decreased.

Cross-sectional data were used, and we analysed the data from a population group within a limited period. In this case, causal relations cannot be certified. More so, as our evidence was limited, the effect of other variables could not be considered. The results are useful in planning a future advanced study.

Given that various specialist physicians request prolonged sick leave, it becomes necessary to raise their awareness to provide complete data, including social and professional aspects and the development of training programs regarding sick leave management. In these programs, two important objectives should be emphasized: improving health conditions and returning to the same social and professional activity level as before. There is also a need to raise awareness of the economic impact of periods of temporary incapacity for work, namely, reduced productivity and costs to the state budget.

It should also be noted that this paper did not analyse information regarding the issue of sick leave certificates for certain diseases (specific cardiovascular diseases, AIDS, neoplasms or tuberculosis). In these cases, according to the current legislative provisions, the allowance for temporary incapacity for work can be granted for a longer period of time, up to 1 year or one and a half years. Additionally, the social insurance physician’s approval is unnecessary; thus, the attending physician has complete responsibility in these cases.

From these perspectives, this paper refers to a limited aspect regarding the temporary incapacity for work and the long-term temporary incapacity for work in well-defined pathological situations (over 183 sick days and requiring the approval of INEMRCM). For a national-level overview of the phenomenon, the present data should be integrated and correlated with more statistical data from other institutions with responsibilities in this field (National Health Insurance House, National House of Public Pensions) and with various socioeconomic data, thereby providing a complete image of the cost of illness and the financial burden of paying sick leave benefits.

## Conclusion

More than three quarters of people who received prolonged sick leave certificates returned to work. The remaining were severely affected, qualifying them to receive a work disability pension, or they met the criteria for early or age-limit retirement. Older people with cardiac or neurological diseases regained their ability to work to a lesser extent. The results of this study show that particular attention is required and individualized intervention measures should be applied to this vulnerable group. This hypothesis should be tested by carrying out further research.

In over 95% of cases, sick leave days were granted, but in practice, it was found that their application did not fully reflect the medical condition but also situations with difficult administrative procedures. Moreover, the burden of sick leave is significant, and findings allow us to draw up some measures to improve these situations.

Sick leave has traditionally been authorized by social insurance physicians and INEMRCM, thereby controlling paid leave expenditures. Recent changes to the Romanian methodology of granting sick leaves have already been made. A new procedure has been established that provides a more thorough assessment of the health condition and monitoring of patients along with a plan for following the evolution of the disease, as drawn up by the attending physician. The rehabilitation plan is customized so that the measures can be closely monitored. The impact of this procedure is expected to be reflected in a decrease in the period of temporary incapacity for work while also achieving full recovery of work capacity and fostering social and professional reintegration.

## Supplementary Information

**Additional file 1.**

## Data Availability

The datasets analysed during the current study are provided in a supplementary file.
